# Genomic and phenotypic characterization of a refactored xylose-utilizing *Saccharomyces cerevisiae* strain for lignocellulosic biofuel production

**DOI:** 10.1186/s13068-018-1269-7

**Published:** 2018-09-29

**Authors:** Phuong Tran Nguyen Hoang, Ja Kyong Ko, Gyeongtaek Gong, Youngsoon Um, Sun-Mi Lee

**Affiliations:** 1 Clean Energy Research Center, Korea Institute of Science and Technology (KIST), Seoul, 02792 Republic of Korea; 2 Division of Energy and Environment Technology, University of Science and Technology (UST), Daejeon, 34113 Republic of Korea; 3 Green School, Korea University, Seoul, 02841 Republic of Korea

**Keywords:** Xylose fermentation, Cofermentation, CRISPR–Cas9, Evolutionary engineering, Gene expression landscape

## Abstract

**Background:**

Engineered strains of *Saccharomyces cerevisiae* have significantly improved the prospects of biorefinery by improving the bioconversion yields in lignocellulosic bioethanol production and expanding the product profiles to include advanced biofuels and chemicals. However, the lignocellulosic biorefinery concept has not been fully applied using engineered strains in which either xylose utilization or advanced biofuel/chemical production pathways have been upgraded separately. Specifically, high-performance xylose-fermenting strains have rarely been employed as advanced biofuel and chemical production platforms and require further engineering to expand their product profiles.

**Results:**

In this study, we refactored a high-performance xylose-fermenting *S. cerevisiae* that could potentially serve as a platform strain for advanced biofuels and biochemical production. Through combinatorial CRISPR–Cas9-mediated rational and evolutionary engineering, we obtained a newly refactored isomerase-based xylose-fermenting strain, XUSE, that demonstrated efficient conversion of xylose into ethanol with a high yield of 0.43 g/g. In addition, XUSE exhibited the simultaneous fermentation of glucose and xylose with negligible glucose inhibition, indicating the potential of this isomerase-based xylose-utilizing strain for lignocellulosic biorefinery. The genomic and transcriptomic analysis of XUSE revealed beneficial mutations and changes in gene expression that are responsible for the enhanced xylose fermentation performance of XUSE.

**Conclusions:**

In this study, we developed a high-performance xylose-fermenting *S. cerevisiae* strain, XUSE, with high ethanol yield and negligible glucose inhibition. Understanding the genomic and transcriptomic characteristics of XUSE revealed isomerase-based engineering strategies for improved xylose fermentation in *S. cerevisiae*. With high xylose fermentation performance and room for further engineering, XUSE could serve as a promising platform strain for lignocellulosic biorefinery.

**Electronic supplementary material:**

The online version of this article (10.1186/s13068-018-1269-7) contains supplementary material, which is available to authorized users.

## Background

The development of xylose-utilizing strains of *Saccharomyces cerevisiae* has improved the prospects of lignocellulosic biorefinery, enabling the creation of full-scale second-generation bioethanol production plants worldwide [1]. However, the main product of xylose-fermenting *S. cerevisiae* strains is generally limited to bioethanol, and further strain engineering is required to expand the product profiles of lignocellulosic biorefinery to include advanced biofuels and chemicals [2]. The primary advantage of using xylose-fermenting strains in lignocellulosic biorefinery is the improvement in the overall bioconversion efficiency [3]. In addition, the unique metabolic characteristics during xylose fermentation, different from those of glucose fermentation, could also place xylose-fermenting strains in a more favorable position for the shift in products from ethanol to advanced biofuels and chemicals. Specifically, the limited accessibility of acetyl-CoA, a central branch point in biosynthetic pathways for advanced biofuels and chemicals, could be resolved in xylose-fermenting strains [2]. Among the engineered strains of *S. cerevisiae*, strains harboring a redox-neutral xylose isomerase-based pathway seem to have a particular advantage over oxidoreductase-based strains, since they introduce no burden in terms of intensive cofactor requirements in the biosynthetic pathways for advanced biofuels and chemicals [4, 5]. Expanding the product profiles of xylose-fermenting *S. cerevisiae*, however, has not been fully explored until recently, since strain engineering has mainly focused on the improvement of xylose fermentation and glucose/xylose cofermentation efficiency [6]. Only a few xylose-fermenting strains have been reported to produce advanced biofuels and chemicals, such as 1-hexadecanol, lactic acid, 2,3-butanediol, and isobutanol, with limited success obtained by introducing the respective synthetic pathways into ordinary xylose-fermenting *S. cerevisiae* strains [7–11]. With recent strain engineering efforts, the xylose fermentation and glucose/xylose cofermentation performance of engineered strains have been greatly improved [6]. However, these high-performance strains, generally developed through plasmid-based integration using auxotrophic markers followed by evolutionary engineering, have rarely been engineered as hosts for advanced biofuel and chemical production [12–15]. The recent development of a refactored oxidoreductase pathway-based xylose-fermenting *S. cerevisiae* strain using the markerless genome-editing tool CRISPR–Cas9 has enabled the development of high-performance xylose-fermenting *S. cerevisiae* producing advanced biofuels and chemicals [16].

In this study, we developed a high-performance xylose-fermenting *S. cerevisiae* strain as a potential production host for advanced biofuels and biochemicals. The introduction of isomerase-based xylose catabolic pathway genes into the *gre3* and *pho13* loci using a markerless genome-editing tool, the CRISPR–Cas9 system, and subsequent evolutionary engineering generated a high-performance xylose-fermenting *S. cerevisiae*, XUSE. The xylose fermentation performance of XUSE was comparable to that of SXA-R2P-E, a representative high-performance xylose-fermenting *S. cerevisiae* strain reported previously [15], and XUSE exhibited adequate cofermentation of glucose and xylose with negligible glucose inhibition. The genomic and transcriptomic analysis of XUSE revealed isomerase-based pathway-specific engineering strategies that could enable further improvement in xylose fermentation performance in terms of ethanol titer, yield and productivity. Consequently, this study provides a promising platform strain of *S. cerevisiae* for advanced biofuel and chemical production from lignocellulosic biomass and offers xylose isomerase pathway-specific engineering strategies for maximizing the xylose fermentation performance of *S. cerevisiae*.

## Results and discussion

### Development of an efficient xylose-fermenting strain of XUSE

**Fig. 1:**
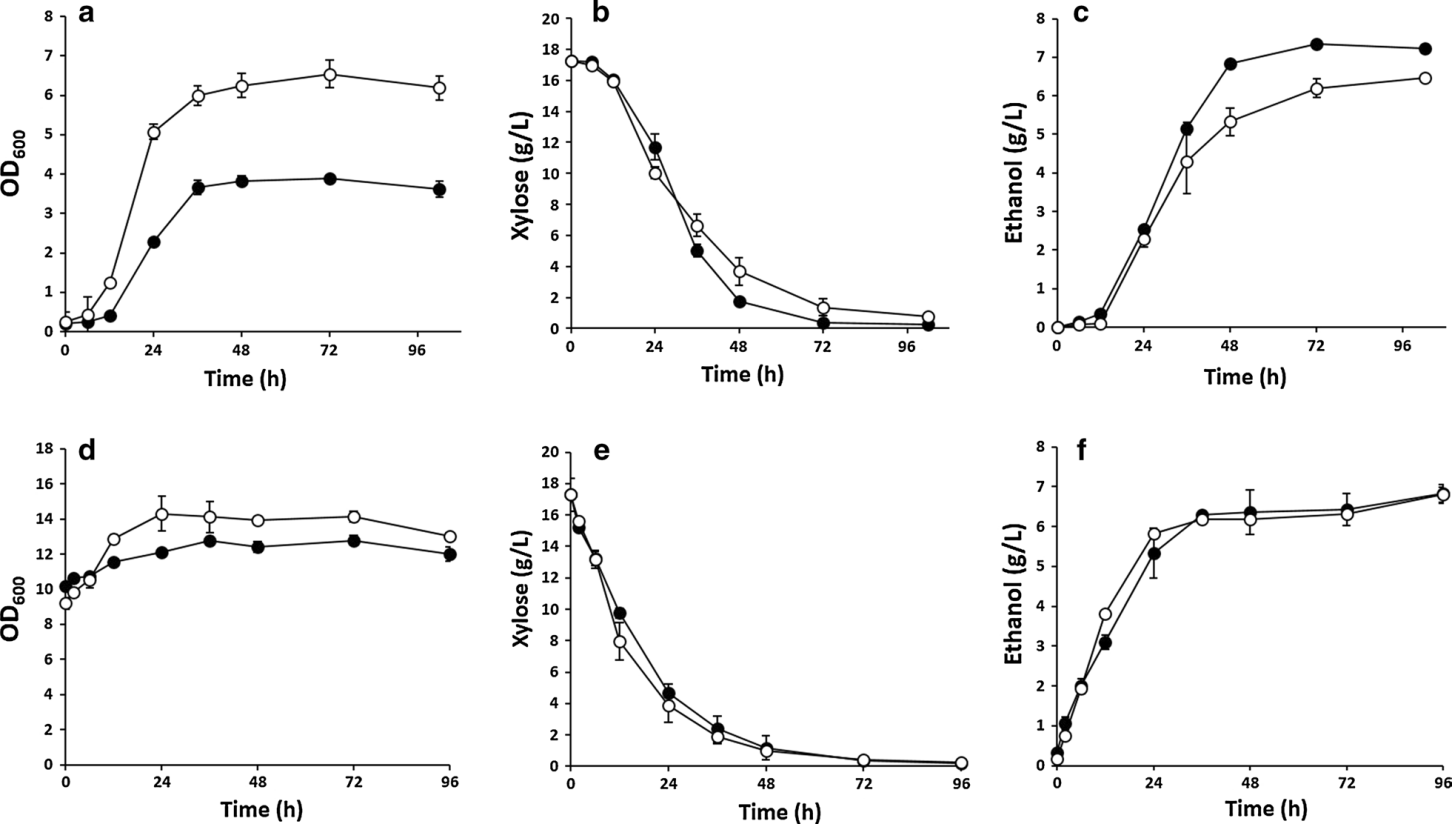
Micro-aerobic fermentation of xylose with XUSE and SXA-R2PE, one of the best xylose-fermenting strains, with an initial OD of **a**–**c** 0.2 and **d**–**f** 10. **a**, **d** Cell growth, **b**, **e** xylose utilization, **c**, **f** ethanol production. Strain identifications (XUSE: ●, SXA-R2PE: ○) are described in the text. Error bars represents standard deviation of biological triplicates

To develop a high-performance xylose-utilizing strain, we sought to refactor one of the best xylose-utilizing strains, SXA-R2P-E [15], using the CRISPR–Cas9 system. To this end, a rationally engineered strain of *S. cerevisiae* was constructed based on the genetic background of SXA-R2P-E (Δ*gre3*, Δ*pho13*, *URA::GPDp*-xylA*3-*CYC1t*-*TEFp*-*XKS1*-*CYC1t*, *Leu::GPDp*-xylA*3-*RPM1t*-*TEFp*-*tal1*-*CYC1t*) [15]. Specifically, xylose isomerase mutant (xylA*3) and xylulokinase (*XKS1*) genes were integrated into the *gre3* loci, and then, an additional copy of the xylose isomerase mutant and transaldolase (*TAL1*) genes was integrated into *pho13* loci to simultaneously integrate and delete the chosen target genes. The rationally engineered strain was then further improved by evolutionary engineering, generating an efficient xylose-fermenting strain of *S. cerevisiae*, XUSE, through combinatorial engineering. XUSE efficiently converted xylose to ethanol, demonstrating comparable xylose fermentation performance to that of SXA-R2P-E (Fig. 1). During 72 h of fermentation, XUSE completely utilized 20 g/L xylose and produced ethanol with a yield of 0.43 g ethanol/g xylose. Interestingly, XUSE generated less cell biomass and produced more ethanol than SXA-R2P-E during low-cell-density fermentation with an initial OD of 0.2, resulting in a slightly higher ethanol yield (0.43 g/g vs 0.4 g/g). This result suggests that the XUSE strain distributes its carbon source more efficiently to ethanol production rather than to cell growth, which would be a beneficial feature in a production host. Similar to SXA-R2P-E, XUSE demonstrated xylose-fermenting performance competitive with that of previously reported strains (Table 1), suggesting successful refactoring to produce a representative xylose-fermenting strain that can be easily engineered for advanced biofuel and biochemical production.

**Table 1:** Comparisons of xylose fermentation performance of the XUSE strain with those of previously reported xylose-fermenting strains

Strain	Genetic background	Fermentation condition		Ethanol yield (g/g)
XUSE (this study)	xylA3*, *TAL1*, *XKS1*, *Δgre3*, *Δpho13,* evolved	Microaerobic batch in serum bottle, synthetic medium (20 g/L xylose)	OD 0.2	0.43
			OD 2	0.45
			OD 10	0.4
SXA-R2P-E	xylA3*, *tal1*, *XKS1*, *Δgre3*, *Δpho13*, evolved	Microaerobic batch in serum bottle, synthetic medium (20 g/L xylose)	OD 0.2	0.4
			OD 2	0.4
			OD 10	0.4
		Anaerobic batch in bioreactor, synthetic medium (40 g/L xylose)		0.45 [15]
RWB217	*xylA*, *TAL1*, *TKL1*, *RPE1*, *RKI1*, *Δgre3*	Anaerobic batch in fermenter, synthetic medium (20 g/L xylose)		0.43 [14]
H131-A3-AL^CS^	*xylA*, *xyl3*, *TAL1*, *TKL1*, *RPE1*, *RKI1*, evolved	Anaerobic batch in bioreactor, synthetic medium (40 g/L xylose)		0.41 [13]
TMB 3424	*xyl1, xyl2, XKS1, TKL1, RPE1, RKI1, ∆gre3,* evolved	Anaerobic batch in bioreactor, synthetic medium (60 g/L xylose)		0.36 [12]

### Evaluation of the glucose/xylose cofermentation performance of XUSE

**Fig. 2:**
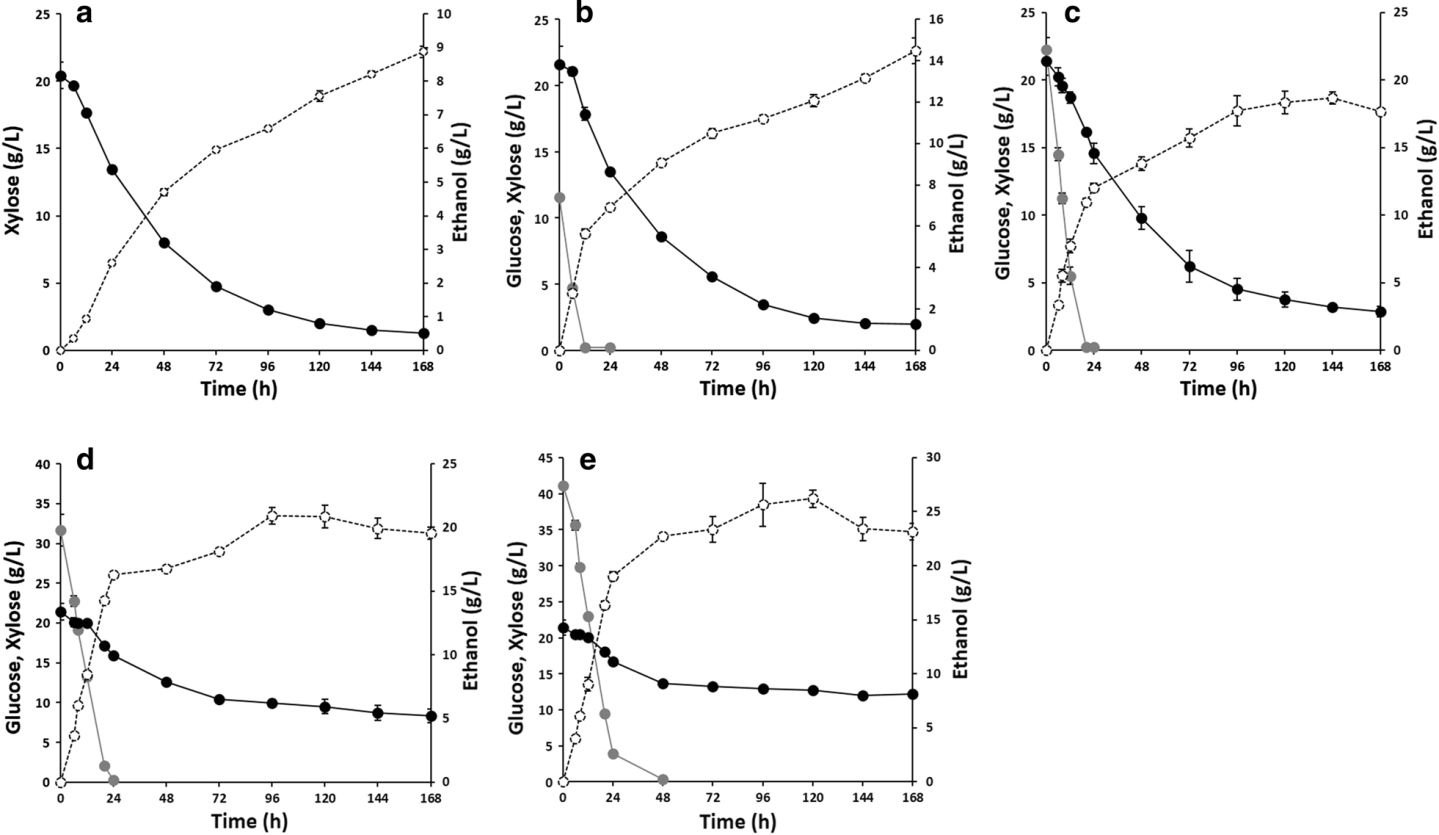
Micro-aerobic cofermentation of glucose and xylose with XUSE strain. Cofermentation was conducted with 20 g/L xylose and varying concentration of glucose (**a** 0 g/L, **b** 10 g/L, **c** 20 g/L, **d** 30 g/L, and **e** 40 g/L). Ethanol production (○, dash line) and xylose (●, solid line), glucose (  , solid line), and utilization profiles were measured during fermentation. Error bars represents standard deviation of biological triplicates

**Fig. 3:**
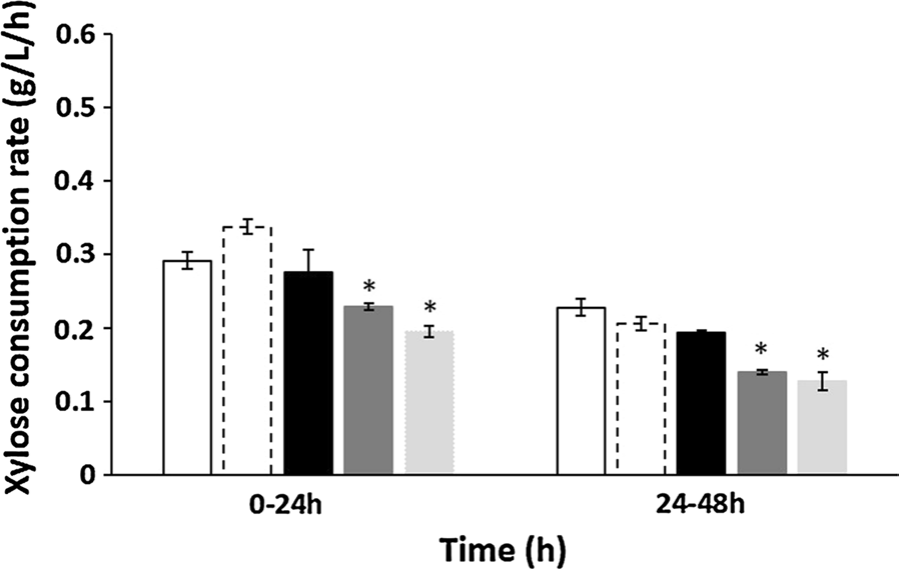
Comparison of xylose consumption rate of XUSE strain throughout glucose/xylose cofermentation with different initial glucose concentrations. Xylose–glucose concentration (g/L) was described as 20–0 (□), 20–10 (  ), 20–20 (  ), 20–30 (  ), and 20–40 (  ). *p < 0.01 vs 20–0

In addition to xylose fermentation performance, the efficient cofermentation of glucose and xylose is also important in developing production hosts for lignocellulosic biorefinery. To evaluate the cofermentation performance of XUSE, we conducted mixed-sugar fermentation of glucose and xylose with varying concentrations of glucose (Fig. 2). When 20 g/L glucose and 20 g/L xylose were supplied, XUSE produced 18.65 g/L ethanol with a yield of 0.46. XUSE exhibited a similar xylose consumption rate during cofermentation to that of SXA-R2P-E (0.2 g/L/h for XUSE vs 0.21 g/L/h for SXA-R2P-E). The total sugar and xylose consumption rates were 0.23 and 0.11 g/L/h, respectively. Interestingly, the xylose consumption rates of XUSE were not significantly affected by the presence of glucose when the glucose concentration was in the range of 0–20 g/L (Fig. 3). Moreover, XUSE simultaneously consumed both xylose and glucose throughout the fermentation process, indicating efficient cofermentation performance (Figs. 2, 3). The xylose consumption rate of XUSE was 0.22, 0.22, and 0.2 g/L/h in the presence of 0, 10, and 20 g/L glucose, respectively. Simultaneous cofermentation has rarely been reported in representative xylose-utilizing *S. cerevisiae* strains. Most xylose-utilizing strains, especially strains with an oxidoreductase pathway, tend to start utilizing xylose only after the complete utilization of glucose (Table 2). The simultaneous cofermentation of glucose and xylose by XUSE indicates the potential of isomerase-based xylose-utilizing strains for lignocellulosic biorefinery, in which the main challenge in cofermentation would not be simultaneous sugar utilization but further improvement of the xylose fermentation efficiency. Although simultaneous coutilization was still observed, the xylose utilization of XUSE was markedly inhibited when the glucose concentration was greater than 30 g/L, suggesting that glucose inhibition should also be relieved, especially at high glucose concentrations.

**Table 2:** Comparison of cofermentation performance of XUSE with those of previously reported xylose-fermenting strains

Strain	Description	Initial glucose–xylose concentration (g/L)	Cofermentation performance			Cofermentation pattern	Refs.
			Sugar consumption rate (g/g/h)	Xylose consumption rate (g/g/h)	Ethanol yield (g/g)		
XUSE	BY4741, xylA*3, TAL1, XKS1, Δgre3, *Δ*pho13, evolved	20–20	0.23	0.11	0.46	A	This study
SXA-R2P-E	BY4741, xylA*3, tal1, XKS1, Δgre3, Δpho13, evolved	20–20	0.17	0.08	0.43	A	[48]
424A (LNH-ST)	Industrial strain, *xyl1, xyl2, XKS1*	65–65	0.26	0.13	0.35	A	[49]
MEC1121	Industrial PE-2, *xyl1, xyl2, XKS1, TAL1*	17–17	0.058	0.029	0.34	A	[50]
S104-TAL	GPY55-15Bα, *xyl1, xyl2, TAL1*	53–53	0.36	0.1	0.3	A	[51]
RBW218	CEN.PK102-3A, *xylA, XKS1, TAL1, RPE1, RKI1, TKL1, ∆gre3,* evolved	20–20	0.51	0.25	0.4	B	[52]
DS68625	DSM, *xylA, XKS1, TAL1, RPE1, RKI1, TKL1, Hxt36*	30–30	0.39	0.18	0.39	B	[53]
Classic-F3	CTY brewing strain, XR, XDH, XKS1	40–40	0.028	0.014	0.38	B	[54]
BP10001	CEN.PK 113-7D, XR, XDH	10–10	0.1	0.034	0.38	B	[55]
MA-R4	IR-2, *xyl1, xyl2, XKS1*	45–45	0.11	0.055	0.38	B	[56]
TMB3400	Industrial USM21	50–50	0.37	0.19	0.36	B	[57]
F106X	YC-DM, *xyl1, xyl2, XKS1, RPE1, RKI1, TKL1, TAL1*	50–50	0.11	0.05	0.36	B	[58]
A4	Industrial strain A, *xyl1, xyl2, XKS1*	50–50	0.13	0.065	0.27	B	[59]
TMB3001	CEN.PK 133-7A, *xyl1, xyl2, XKS1*	50–50	0.11	0.037	0.23	B	[59]

Cofermentation pattern A: simultaneous cofermentation, B: sequential cofermentation

### Significant nucleotide changes derived from evolutionary engineering improved the xylose fermentation performance of the XUSE strain

**Fig. 4:**
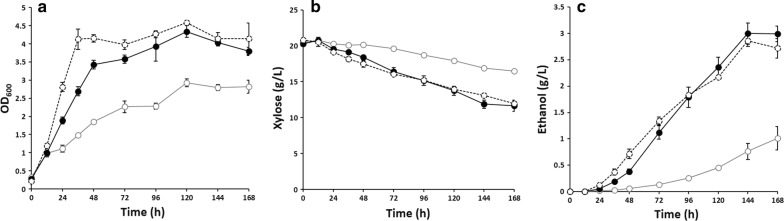
Effect of *PMR1* mutation on xylose fermentation performance. Xylose fermentation was conducted with *PMR1* deletion strains harboring wild-type *PMR1* (○, solid line), mutated *PMR1* (●, solid line), or empty plasmid (○, dash line). **a** Cell growth, **b** xylose utilization, and **c** ethanol production

**Fig. 5:**
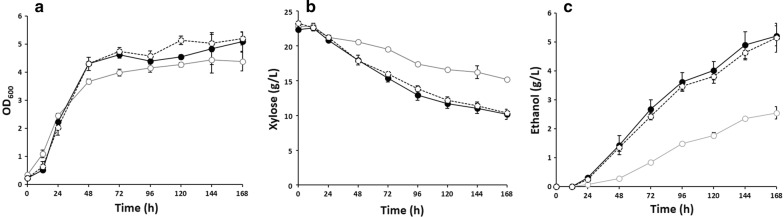
Effect of *ASC1* mutation on xylose fermentation performance. Xylose fermentation was conducted with *ASC1* deletion strains harboring wild-type *ASC1* (○, solid line), mutated *ASC1* (●, solid line), or empty plasmid (○, dash line). **a** Cell growth, **b** xylose utilization, and **c** ethanol production

To investigate whether beneficial mutations contributed to the improved xylose fermentation performance of XUSE, we conducted whole-genome sequencing analysis and identified two mutations in YGL167C (*PMR1*^*G681A*^) and YMR116C (*ASC1*^Q237*^). The mutation and deletion of *PMR1*, encoding a Golgi Ca^2+^/Mg^2+^ ATPase [17], have previously been reported to improve xylose isomerase activity and anaerobic growth on xylose [18, 19]. ASC1, which encodes the G-protein beta subunit and guanine dissociation inhibitor for Gpa2p, has been reported to be involved in the glucose-mediated signaling pathway and invasive growth in response to glucose limitation [20], but little is known about its effect on xylose fermentation. The beneficial effects of the identified mutations on xylose fermentation were then confirmed through complementation experiments (Figs. 4, 5). When the mutated genes were expressed in the respective knockout strains (P11 and P12 strains; Additional file 1: Table S1), xylose consumption and ethanol production were significantly improved, indicating that the evolutionary engineering-derived point mutations in *PMR1* and *ASC1* contributed to the improved xylose utilization in XUSE. The strains expressing *Pmr1*^*G681A*^ and *Asc1*^Q237*^ consumed 114.8% and 59.6% more xylose and produced 195.9% and 104.4% more ethanol than the strain with the respective wild-type genes (Figs. 4,  5). Interestingly, *PMR1* and *ASC1* knockout strains exhibited similar fermentation performance to the strains harboring the respective genes with identified mutations, suggesting that the given mutations lead to loss of gene function. A single amino acid change (Pmr1^G249V^) or the deletion of *PMR1* was previously reported to improve xylose utilization in *S. cerevisiae* [19]. Although the newly identified mutation in *PMR1* (Pmr1^*G681A*^) is different from the one identified in the previous study, the beneficial impact is still correlative. Although little is known about the effects of the deletion or malfunction of *ASC1* on xylose metabolism in *S. cerevisiae*, inactivation of *ASC1* was previously reported to be beneficial for the cell growth of *S. cerevisiae* under oxygen-depletion conditions [21]. In addition, Asc1p is known as a negative regulator of various metabolic and signal transduction pathways [22] and specifically represses Gcn4p. Gcn4p has been reported to be a representative transcription factor regulating the genes involved in xylose metabolism in efficient xylose-fermenting strains [23]. Therefore, *ASC1* malfunction seems to potentially improve cell growth under microaerobic fermentation conditions and enhance the expression of genes involved in xylose metabolism.

### The gene expression landscape of XUSE revealed engineering strategies for enhanced xylose metabolism in *S. cerevisiae*

**Fig. 6:**
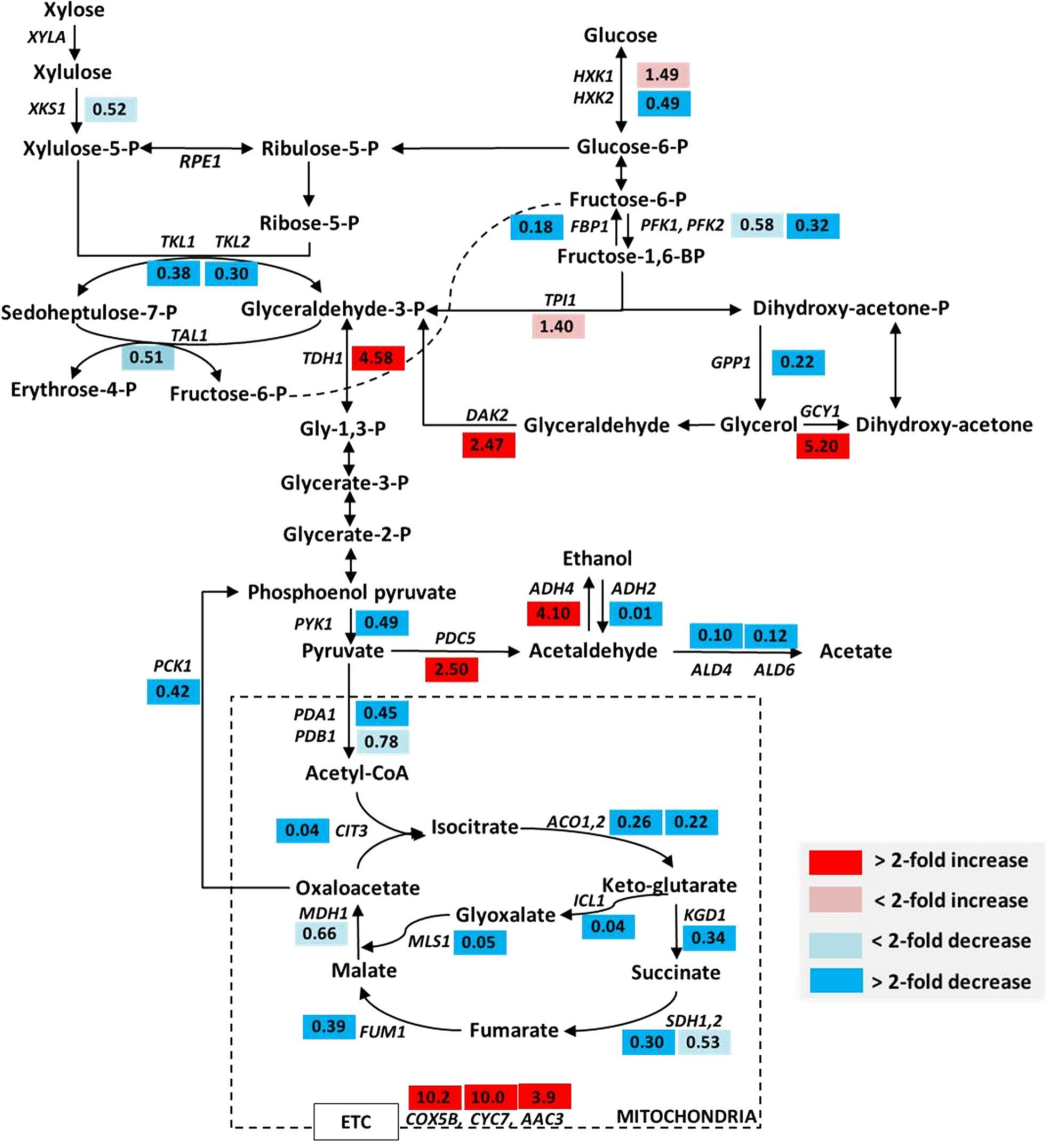
Comparison of transcription levels of the genes involved in xylose metabolic pathways and central carbon metabolism of XUSE and XUS strains. The cells were cultured in minimal medium containing 20 g/L xylose under microaerobic conditions and then collected after 20 h of cultivation. The values in the boxes indicate the fold changes of transcription levels in XUSE compared with those in XUS (*p* < 0.05). The gene names follow those in the *Saccharomyces* Genome Database (SGD; http://www.yeastgenome.org)

To understand the mechanisms of the enhanced xylose metabolism of XUSE, the global transcript profiles of XUS (rationally engineered strain) and XUSE (evolved XUS strain) grown on xylose were analyzed. Compared with XUS, XUSE showed a significantly different gene expression landscapes, with 463 upregulated and 675 downregulated genes (> 2-fold). The transcriptional changes in genes involved in the central carbon metabolism are presented on the respective metabolic pathway map (Fig. 6).

Upon profiling of the differentially expressed genes, we noted a general pattern: the genes involved in the nonoxidative pentose phosphate pathway, such as *TKL1*, *TKL2,* and *TAL1*, were downregulated in XUSE (Fig. 6 and Table 3). This result suggests that evolutionary engineering fine-tuned the xylose metabolic flux by repressing PP pathway enzymes and that the overexpression of PP pathway genes is not a prerequisite for improved xylose fermentation performance in *S. cerevisiae*. This result partially explains the high performance of the minimally engineered XUSE, in which the PP pathway genes are not overexpressed. Of the PP pathway genes, the *TKL2* gene was most strongly downregulated in XUSE, by 3.3-fold (Table 3), and its disruption was previously verified to have a positive effect on xylose fermentation [24].

**Table 3:** Fold changes (XUS vs XUSE) in the expression of the genes involved in xylose metabolism

Gene	ORF	Fold change in expression^a^	Function	Pathway
*XKS1*	YGR194C	0.52	Xylulokinase	PP pathway
*TAL1*	YLR354C	0.51	Transaldolase	PP pathway
*TKL1*	YPR074C	0.38	Transketolase	PP pathway
*TKL2*	YBR117C	0.30	Transketolase	PP pathway
*PMR1*	YGL167C	0.59	High-affinity Ca^2+^/Mn^2+^ ATPase	Transportation of Ca^2+^ and Mn^2+^ ions into Golgi apparatus
*ISU1*	YPL135W	0.34	Iron–sulfur cluster scaffold protein	Scaffolding function during assembly of iron–sulfur cluster

^a^Fold change is the ratio of the transcription level in the evolved cells (XUSE) to that in the control cells (XUS) (*p* < 0.05)

The genes involved in the regulation of cellular metal ion concentrations, such as *PMR1, PHO84,* and *ISU1*, showed significantly changed expression levels in XUSE (Table 3 and Additional file 2: Table S2). The downregulation of *PMR1* expression, impeding manganese ion export from the cell through the secretory pathway, corresponds to loss-of-function mutations (Fig. 4). In contrast, *PHO84,* which encodes an inorganic phosphate transporter with a major role in manganese homeostasis [25], was upregulated by 3.7-fold in XUSE (Additional file 2: Table S2). Therefore, manganese ions accumulated in the cells via this transporter could be more easily incorporated into manganese-requiring enzymes such as xylose isomerase [25]. In addition to manganese ions, the increased cellular iron cation concentration resulting from *ISU1* downregulation (0.34-fold, *p *< 0.05) could also increase xylose isomerase activity and other cellular processes beneficial for xylose metabolism [26]. Although iron cations are not preferred by xylose isomerase, they play essential roles as cofactors for several cellular processes and have been reported to boost *xylA* activity [26, 27]. Specifically, Santos et al. [26] found that inactivation of the *ISU1* gene, which encodes a scaffold protein involved in the assembly of iron–sulfur clusters, occurred during adaptive evolution and improved xylose fermentation efficiency. Therefore, the fine-tuned cellular metal ion concentrations in XUSE could have led to improved xylose fermentation performance by boosting *xylA* activity and other cellular processes (Table 3).

**Fig. 7:**
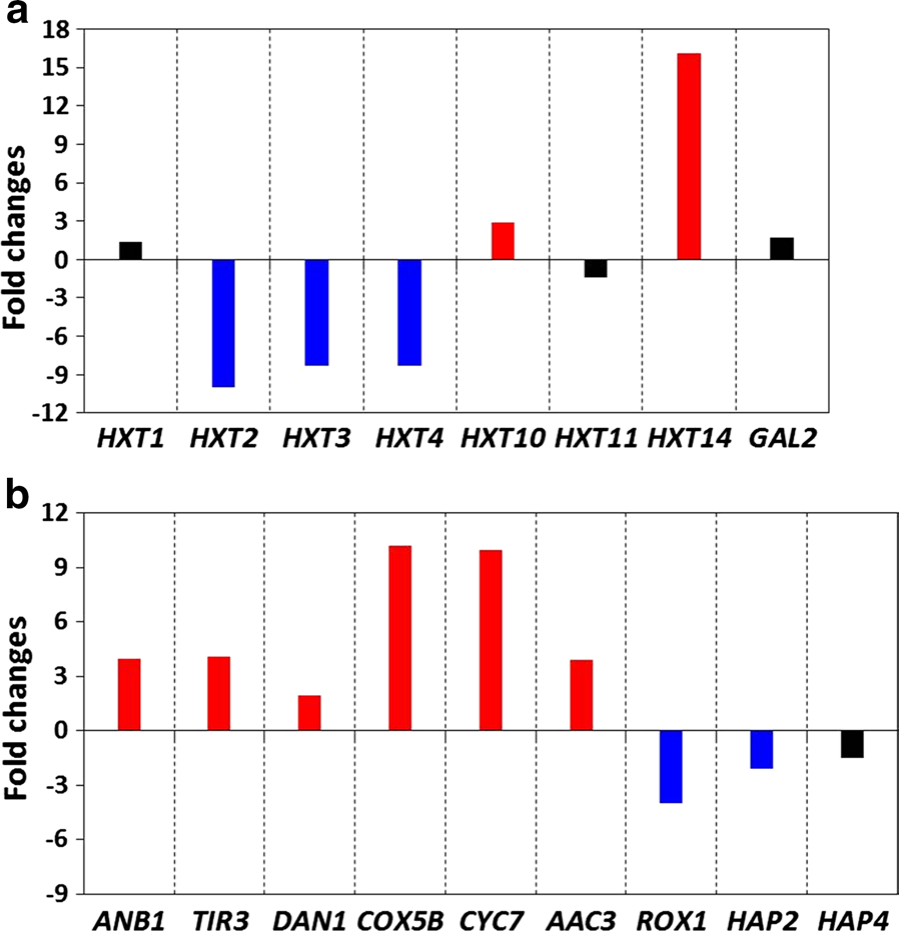
Fold changes (*p* < 0.05) in the expression of **a** sugar transporters and **b** hypoxic genes (*ANB1*, *TIR3*, *DAN1*, *COX5B*, *CYC7*, and *AAC3*) and their transcriptional repressors *(ROX1, HAP2,* and *HAP4*) in XUSE compared with expression in the XUS strain

Through evolutionary engineering, the transcriptomic landscape of hexose transporters (*HXT1*–*17* and *GAL2*) was changed to significantly increase *HXT14* expression and to greatly decrease the expression of *HXT2* and *HXT4* (Fig. 7a). In accordance with *HXT14*, whose expression was increased exceptionally by 16-fold, the transcription of *HXT10*, *GAL2*, *HXT8*, *HXT1*, and *HXT9* increased by 2.9-, 1.7-, 1.5-, 1.4-, and 1.1-fold, respectively. Interestingly, these hexose transporters have previously been shown to be more closely related to xylose-preferred sugar transporters based on evolutionary distances in terms of the G-G/F-XXXG motif among native sugar transporters of *S. cerevisiae* [28]. In contrast, the most downregulated *HXT2* and *HXT4* are known to be glucose-preferring hexose transporters with longer evolutionary distances in terms of the G-G/F-XXXG motif [28]. These changes in the transcriptomic profiles of sugar transporters could partially explain the improved xylose fermentation performance with the simultaneous cofermentation of glucose and xylose by XUSE. Until recently, studies have focused on the engineering of *HXT1*–*HXT7* to boost the efficiency of xylose utilization [6, 29]. The transcriptome profiles of XUSE and recent reports on the beneficial roles of HXT11 [30] and HXT14 [31] in improving xylose fermentation performance, however, suggest the need to engineer underexplored sugar transporters to develop xylose-utilizing *S. cerevisiae* at more advanced levels.

Several genes, including *COX5B*, *CYC7*, *AAC3*, *ANB1,* and *TIR3*, which are predominantly induced during anaerobic/hypoxic growth [32, 33], were significantly upregulated in the evolved strain (Fig. 7b and Additional file 2: Table S2). *ANB1* (YJR047C), which encodes the translation elongation factor eIF-5A [34], was expressed at a fourfold higher level in XUSE than in XUS. *COX5B* and *CYC7*, which are responsible for respiration under anaerobic/hypoxic conditions, were highly upregulated by tenfold (Fig. 7b and Additional file 2: Table S2), while most of the genes associated with the TCA cycle and aerobic respiration were repressed in XUSE (Fig. 6 and Additional file 2: Table S2). Moreover, several transcriptional repressors (*ROX1*, *HAP2,* and *HAP4*) of anaerobic respiratory genes were also decreased by 1.5–4-fold in XUSE (Fig. 7b) [35]. These results suggest that the XUSE strain seemed to have taken an evolutionary path toward enhanced anaerobic growth, thus improving anaerobic fermentation performance with an ethanol yield of 0.43 g/g.

In the ethanol fermentation pathway, enzyme transcripts for ethanol oxidation (encoded by *ADH2*) decreased significantly in the XUSE strain, while those of *ADH4* and *PDC5,* encoding alcohol dehydrogenase and pyruvate decarboxylase, respectively, increased by 2.5–4-fold (Fig. 6). Among the aldehyde dehydrogenase genes involved in acetate formation, the expression levels of *ALD4* and *ALD6* were greatly decreased by 8–10-fold. The transcriptional changes in ethanol fermentation and acetate formation pathways could also contribute to the increased metabolic flux toward ethanol production in the XUSE strain.

Among the top 20 transcription factors affecting the highest numbers of genes involved in xylose regulation [23], the stress-responsive transcription factors *MSN2* and *MSN4* showed significantly changed expression levels. Specifically, *MSN*2 was observed to be downregulated by 2.8-fold, while its homolog *MSN4* was upregulated in the evolved strain (2.4-fold, *p* < 0.05) [36] (Additional file 3: Table S3). This result is consistent with the report by Matsushika et al. [36], in which the expression of *MSN4* was induced, while *MSN2* transcription was comparable during xylose fermentation and glucose fermentation. In agreement with the increased transcription of *MSN4*, the transcription of known target genes of MSN2/4, such as *DDR2, GSY2*, *ALD2*, *ALD3,* and *CTT1*, was upregulated. Interestingly, however, some MSN2/4 target genes of *SSA3* and *TKL2* were repressed, suggesting a higher influence of MSN2 on the regulation of these genes. Although MSN2/4 is known to be functionally redundant, gene- and stress condition-specific regulatory contributions of MSN2/4 have been previously reported, supporting the downregulated gene expression of the MSN2/4-dependent genes of *SSA3* and *TKL2* (Additional file 3: Table S3) [37, 38]. Another stress response protein of HSP30, reported to be independent of *MSN*2*/4* [39], was greatly induced (by 32-fold) in XUSE (Additional file 2: Table S2). Since HSP30 acts as a negative regulator of the H^+^-ATPase Pma1 pump, its induction could have led to the downregulation of the stress-stimulated H^+^-ATPase [40]. This result suggests that the adaptation of the XUSE strain to glucose limitation or other energy-demanding stresses may limit ATP usage by ATPase, thus decreasing the energy requirement for maintenance during xylose fermentation [40].

### Evolutionary paths toward the enhanced xylose fermentation of XI-based strains

To understand the evolutionary trajectories of isomerase-based xylose-fermenting strains, the transcriptional landscapes of evolved xylose-fermenting strains harboring redox-neutral xylose isomerase and cofactor-imbalanced oxidoreductase-based pathways (XI-based strains and XR/XDH-based strains, respectively) were compared (Additional file 4: Figure S1). The most pronounced difference between the XI- and XR/XDH-based strains was the transcription levels of the genes involved in the nonoxidative PP pathway. As shown in Additional file 4: Figure S1, nonoxidative PP pathway enzymes were downregulated (or rearranged) to reduce their transcriptional burdens in XI-based strains [24, 41]. Since the limited metabolic flux through the PP pathway is a known bottleneck for efficient xylose fermentation, strain engineering for enhanced xylose metabolism often involves overexpression of the genes in the nonoxidative PP pathway [15, 24]. Although *XKS1* and *TAL1* were rationally overexpressed in the XUS strain, evolutionary engineering led to the downregulation of their transcription to maintain the balance of the PP and glycolytic pathways (Table 3). In agreement with the results of this study, Qi et al. [24] reported that the evolutionary process repressed the transcription of *TKL1* and *TKL2* to optimize xylose metabolism in the XI-based recombinant strain. However, XR/XDH-based strains have evolved to increase the expression levels of *XYL1*, *XYL2*, *XKS1,* and both oxidative and nonoxidative PP pathway genes (*TKL1, TAL1, SOL1, SOL3,* and *GND1*) [42, 43]. The cellular responses distinctively support a newly introduced xylose assimilation pathway. Whereas XR/XDH-based strains differentially express the genes involved in redox metabolism, because the first two steps of xylose utilization impose an anaerobic redox imbalance [42], XI-based strains show genomic and transcriptomic changes associated with metal homeostasis to support maximal XI activity (Additional file 4: Figure S1b). Specifically, while the XR/XDH strains tend to show higher expression levels of redox balance-related genes (e.g., *NDE1*, *ZWF1,* and *GND2*) on xylose than on glucose [44, 45], significant changes in those genes were not observed in XI-based strains. As distinguishing characteristics that are seemingly necessary for microaerobic growth of the XI pathway-based strains on xylose, the decreased transcription of a number of genes encoding the TCA cycle and respiratory enzymes was observed only in XI-based strains (Fig. 6) (this study and [24]). The changes in the transcript levels of respiratory enzymes imply that the evolved strains exhibit anaerobic characteristics and require a lower level of maintenance energy during cell growth on xylose than the original strains [24, 45]. One suggestion is that reducing the maintenance energy requirement of the xylose-metabolizing strains is crucial for improving xylose-based ethanol production [23, 45].

## Conclusions

In this study, we successfully developed a high-performance xylose-fermenting strain of *S. cerevisiae,* XUSE, through CRISPR–Cas9-mediated rational engineering and evolutionary engineering. XUSE exhibited comparable xylose fermentation performance to that of SXA-R2P-E, one of the best xylose-fermenting *S. cerevisiae* strains, with good cofermentation of glucose and xylose. Genomic and transcriptomic analysis of XUSE uncovered a new engineering target, *ASC1,* and provided isomerase-based strain-specific engineering strategies to further improve xylose utilization in *S. cerevisiae*. With room for further engineering, XUSE could serve as a promising platform strain for lignocellulosic biorefinery.

## Methods

### Strains and culture conditions

All strains and plasmids used in this study are listed in Additional file 1: Table S1. *S. cerevisiae* strain BY4741 was used as a host strain and routinely cultivated at 30 °C in yeast synthetic complete (YSC) medium including 20 g/L glucose (or xylose), 6.7 g/L yeast nitrogen base (Difco, Detroit, MI, USA), and CSM–His–Ura (complete synthetic medium without His and Ura) or CSM (MP Biomedicals, Solon, Ohio, USA). *E. coli* strain D10β was used for cloning and plasmid harvest and cultured at 37 °C in Luria–Bertani (LB) broth supplemented with 100 µg/mL ampicillin. All cultivations were performed in orbital shakers at 200 rpm.

### Analytical methods

Cell growth was analyzed by measuring the optical density at 600 nm using a Cary 60 Bio UV–Vis Spectrophotometer (Agilent Technologies, USA). The concentration of glucose and xylose was quantified by a high-performance liquid chromatography system (HPLC 1260 Infinity, Agilent Technologies, CA, USA) equipped with a refractive index detector (RID) and using a Hi-Plex H column (Agilent Technologies, Palo Alto, CA, USA) under the following conditions: 5 mM H_2_SO_4_ as the mobile phase, a flow rate of 0.6 mL/min, and a column temperature of 65 °C. The ethanol concentration was determined by a gas chromatography (GC) system equipped with a flame ionization detector (FID) using an HP-INNOWax polyethylene glycol column (30 m × 0.25 µm × 0.25 µm) (Agilent Technologies, CA, USA).

### Construction of xylose-utilizing strain using CRISPR–Cas9

To construct the rationally engineered *S. cerevisiae* strain, CRISPR–Cas9-based gene integration was performed using the plasmids listed in Additional file 1: Table S1. The CRISPR–Cas9 system was slightly modified from that in a previous paper [46] as follows. (i) p413-Cas9 was constructed from the p414-TEF1p-Cas9-CYC1t plasmid (Addgene plasmid #43802). (ii) The gRNA expression plasmid (p426gGRE3 or p426gPHO13) targeting *GRE3* (GCCCGGTACGTATCTATGAT) or *PHO13* (TTCAATCATGGAGCCTGCAC) was modified by replacing the target sequence of the previous gRNA expression plasmid (Addgene #43803) [46] (Additional file 1: Table S1). The p413-Cas9 plasmid was first cotransformed with a p426PHO13 plasmid and donor DNA fragments containing an overexpression cassette of xylA3* [47] and *XKS1* (GPDp-xylA3*-PRM9t-TEFp-*XKS1*-CYC1t) into the BY4741 strain using a Frozen EZ Yeast Transformation II Kit (Zymo Research, Irvine, CA, USA). Then, an additional copy of each of xylA3* and *TAL1* (GPDp-xylA3*-PRM9t-TEFp-*TAL1*-CYC1t) was integrated into the *gre3* locus by cotransforming p413-Cas9 and p426gGRE3, resulting in the XUS strain. The final strain (XUS) was subjected to subculture on CSM supplemented with 20 g/L glucose for plasmid rescue after integration was verified by PCR-based diagnosis.

### Evolutionary engineering of xylose-utilizing strain

To improve the xylose utilization of the rationally engineered strain (XUS), evolutionary engineering was applied by subculturing in 50 mL falcon tubes containing CSM medium supplemented with 20 g/L xylose with an initial OD_600_ of 0.2. To achieve the most effective and fastest growth selection, cells were serially transferred into fresh medium at the exponential phase (OD_600_ from 2 to 2.5) using 0.5% inoculum in biological triplicates [15]. After nine rounds of subcultures, the 100 largest colonies were isolated. The cell growth of the isolated variants was first evaluated using TECAN Infinite Pro 200 (Tecan Group Ltd., Männedorf, Switzerland), and they were then screened in 3 mL of CSM medium with 20 g/L xylose in a 14 mL culture tube. Finally, the fastest growing strain, XUSE, was selected in fermentation experiments using serum bottles under microaerobic conditions.

### Phenotypic characterization of the XUSE strain

The XUSE strain was phenotypically characterized by its xylose fermentation and cofermentation performance. For fermentation, seed culture was prepared in YSC medium containing 20 g/L glucose by inoculation with a glycerol stock. Cells were then transferred to fresh YSC medium containing xylose or xylose/glucose as carbon sources and incubated aerobically at 30 °C for 1.5 days for preculture. The precultured cells were then transferred into YSC medium (pH 5.0) containing 100 mM phthalate buffer (pH 5.0). The microaerobic fermentations were conducted in 250 mL serum bottles with an initial OD_600_ of 0.2, 2, or 10 at 30 °C with orbital shaking at 200 rpm. The serum bottles containing 40 mL of media were capped with rubber stoppers, which were pierced with a needle.

### Genotypic characterization of the XUSE strain

The genomic DNA of the XUS and XUSE strains was extracted using the Wizard Genomic DNA Purification Kit (Promega, WI, USA). Whole-genome sequencing was performed using an Illumina HiSeq 2500 platform by the service from Macrogen, Inc., South Korea. The whole-genome sequences of XUS and XUSE strains were compared to discover genetic changes in XUSE, and several genes of interest were selected and verified by Sanger sequencing. In the evolved strain, we identified the genes *PMR1* and *ASC1* those might be responsible for improved xylose utilization. For the functional analysis of the identified mutations in *PMR1* and *ASC1*, the P11 (P1 *∆pmr1*) and P12 (P1 *∆asc1*) strains derived from the P1 strain (BY4741 *∆gre3 ∆pho13*) by the CRISPR–Cas9 editing system were used to construct the P111, P112, P113, P121, P122, and P123 strains, as listed in Additional file 1: Table S1.

### Transcriptomic characterization of the XUSE strain

RNA-sequencing analyses were performed using tools from the commercial RNA-Seq service Ebiogen, Inc. (Seoul, Republic of Korea). After 20 h microaerobic fermentations with xylose as the sole carbon source, yeast cells were harvested by centrifugation at 500×*g* and 4 °C for 5 min. Total RNA was extracted using Trizol reagent (Invitrogen, CA, USA) according to the manufacturer’s protocol provided by Ebiogen, Inc. (Seoul, Republic of Korea). Each of the total RNA samples was evaluated for RNA quality control based on the 28S/18S ratio and RIN measured on the 2100 Bioanalyzer system (Agilent Technologies, Waldbronn, Germany). The cDNA library was constructed using the Clontech SMARTer Stranded RNA-Seq kit (Clontech, Mountain View, CA, USA). High-throughput sequencing was performed on an Illumina HiSeq 2500 system (Illumina, Inc., San Diego, CA, USA). The raw sequence data of XUS and XUSE have been deposited in NCBI’s Gene Expression Omnibus and are accessible through GEO Series Accession Number GSE116076.

## Additional files

**Additional file 1: Table S1.** Strains and plasmids used in this study.

**Additional file 2: Table S2.** Metabolism-related genes with significantly changed expression levels in XUSE relative to those in XUS.

**Additional file 3: Table S3.** Expression levels of known target genes dependent on Msn2/4p.

**Additional file 4: Figure S1.** Comparison of the trends in the transcriptional changes in XI-based (a) and XR/XDH-based (b) strains during adaptive evolution on xylose.
