# Supplementary material for: Genomic and phenotypic characterization of a refactored xylose-utilizing Saccharomyces cerevisiae strain for lignocellulosic biofuel production
Source: Biotechnol Biofuels. 2018 Sep 29;11:268. doi: 10.1186/s13068-018-1269-7 (PMC6162923; doi:10.1186/s13068-018-1269-7)
Supplement: Supplementary file 1 — Additional file 1: Table S1. Strains and plasmids used in this study. [file 13068_2018_1269_MOESM1_ESM.docx]

**Table S1.** Strains and plasmids used in this study.

| Strains | Characteristics | References |
| --- | --- | --- |
| *S. cerevisiae* BY4741 | *MATa his3Δ1 leu2Δ0 met15Δ0 ura3Δ0* | [60] |
| XUS | BY4741 *gre3::GPDp-xylA*3-PRM9t-TEFp-XKS1-CYC1t*, *pho13::GPDp-xylA*3-PRM9t-TEFp-TAL1-CYC1t* | This study |
| XUSE | Evolved strain of XUS | This study |
| SXA-R2P-E | BY4741 *∆gre3*, *URA::GPDp-xylA*3-CYC1t-TEFp-XKS1-CYC1t*, *Leu::GPDp-xylA*3-RPM1t-TEFp-tal1-CYC1t*, *YDL236w::His*, evolved | [15] |
| P1 | BY4741 *∆gre3*, *∆pho13* | This study |
| P11 | P1 *∆pmr1* | This study |
| P12 | P1 *∆asc1* | This study |
| P111 | P11 p416xylA-XKS1, p413xylA-TAL1, pWT-PMR1 | This study |
| P112 | P11 p416xylA-XKS1, p413xylA-TAL1, pMT-PMR1 | This study |
| P113 | P11 p416XylA-XKS1, p413xylA-TAL1, p415 TEFp-CYC1t | This study |
| P121 | P12 p416xylA-XKS1, p413xylA-TAL1, pWT-ASC1 | This study |
| P122 | P12 p416xylA-XKS1, p413xylA-TAL1, pMT-ASC1 | This study |
| P123 | P12 p416xylA-XKS1, p413xylA-TAL1, p415 TEFp-CYC1t | This study |
| Plasmids | Characteristics | References |
| p413Cas9 | P413 TEFp- Cas9- CYC1t | This study |
| p426gGRE3 | p426 SNR52p-gRNA.GRE3.28.392-SUP4t targeting *GRE3* | This study |
| p426gPHO13 | p426 SNR52p-gRNA.PHO13.28.561-SUP4t targeting *PHO13* | This study |
| p416XylA-XKS1 | p416 GPDp-xylA*3-PRM9t-TEFp-XKS1-CYC1t | This study |
| p413XylA-TAL1 | p413 GPDp-xylA*3-PRM9t-TEFp-tal1-CYC1t | This study |
| pWT-PMR1 | p415 TEFp-wtPMR1-CYC1t | This study |
| pMT-PMR1 | p415 TEFp-mtPMR1-CYC1t | This study |
| pWT-ASC1 | p415 TEFp-wtASC1-CYC1t | This study |
| pMT-ASC1 | p415 TEFp-mtASC1-CYC1t | This study |

**Reference**

60. Brachmann CB, Davies A, Cost GJ, Caputo E, Li J, Hieter P, Boeke JD. Designer deletion strains derived from *Saccharomyces cerevisiae* S288C: A useful set of strains and plasmids for PCR-mediated gene disruption and other applications*.* Yeast. 1998;14:115-32.
