# Supplementary material for: Genomic and phenotypic characterization of a refactored xylose-utilizing Saccharomyces cerevisiae strain for lignocellulosic biofuel production
Source: Biotechnol Biofuels. 2018 Sep 29;11:268. doi: 10.1186/s13068-018-1269-7 (PMC6162923; doi:10.1186/s13068-018-1269-7)
Supplement: Supplementary file 2 — Additional file 2: Table S2. Metabolism-related genes with significantly changed expression levels in XUSE relative to those in XUS. [file 13068_2018_1269_MOESM2_ESM.docx]

**Table S2:** Metabolism-related genes with significantly changed expression levels in XUSE relative to those in XUS.

| Gene name | Fold change in expression^a^ | Function |
| --- | --- | --- |
| Glycolysis | | |
| *HXK1* | 1.5 | Hexokinase |
| *HXK2* | 0.49 | Hexokinase |
| *TDH1* | 4.6 | Glyceraldehyde-3-phosphate dehydrogenase |
| *TPI1* | 1.4 | Triose phosphate isomerase |
| *PFK1/PFK2* | 0.58/0.32 | Phosphofructokinase |
| *PYK1* | 0.49 | Pyruvate kinase |
| *PDA1* | 0.45 | Pyruvate dehydrogenase |
| Gluconeogenesis | | |
| *FBP1* | 0.18 | Fructose-1,6-bisphosphatase |
| *PCK1* | 0.42 | Phosphoenolpyruvate carboxykinase |
| *MDH2* | 0.26 | Malate dehydrogenase |
| *GDH2* | 0.22 | Glutamate dehydrogenase |
| *ICL1* | 0.04 | Isocitrate lyase |
| Electron transport chain | | |
| *COX5B* | 10.2 | Cytochrome c oxidase |
| *CYC7* | 10.0 | Cytochrome c isoform 2 |
| *SDH5* | 4.0 | Protein required for flavinylation of Sdh1p |
| *AAC3* | 3.9 | Mitochondrial ADP/ATP translocator |
| *RSF1* | 3.4 | Protein required for respiratory growth |
| *HAP4* | 0.68 | Transcriptional activator of respiratory gene expression |
| TCA cycle | | |
| *CIT3* | 0.04 | Mitochondrial citrate and methylcitrate synthase |
| *ICL1* | 0.04 | Isocitrate lyase |
| *MLS1* | 0.05 | Malate synthase |
| *IDP2* | 0.15 | Cytosolic NADP-specific isocitrate dehydrogenase |
| *ACO1/ACO2* | 0.26/0.22 | Mitochondrial aconitase |
| *KGD1* | 0.34 | Subunit of mitochondrial α-ketoglutarate dehydrogenase |
| *SDH1/SDH2* | 0.13/0.53 | Subunit of succinate dehydrogenase |
| *FUM1* | 0.39 | Fumarase |
| *MDH1* | 0.66 | Mitochondrial malate dehydrogenase |
| Phosphate utilization/regulation | | |
| *SPL2* | 10.4 | Protein with similarity to cyclin-dependent kinase inhibitors |
| *GIT1* | 5.2 | Plasma membrane permease |
| *PHO84* | 3.7 | High-affinity inorganic phosphate transporter |
| *PHO12* | 2.3 | One of three repressible acid phosphatases |
| *PHO11* | 2.2 | One of three repressible acid phosphatases |
| Other | | |
| *EGO4* | 35.1 | Unknown function |
| *HSP30* | 32 | Negative regulator of the H(+)-ATPase Pma1p |
| *FIG1* | 18.8 | Integral membrane protein required for efficient mating |
| *GCY1* | 5.2 | Glycerol dehydrogenase |
| *TIR3* | 4.1 | Cell wall mannoprotein |
| *GSY1* | 4.1 | Glycogen synthase |
| *ANB1* | 4.0 | Translation elongation factor eIF-5A |
| *DAN1* | 2.0 | Cell wall mannoprotein |
| *MIS1* | 0.56 | C1-tetrahydrofolate synthase |

^a^Fold change is the ratio of the transcription level in evolved cells (XUSE) to that in control cells (XUS) (*p* < 0.05).
