# Supplementary material for: Genomic and phenotypic characterization of a refactored xylose-utilizing Saccharomyces cerevisiae strain for lignocellulosic biofuel production
Source: Biotechnol Biofuels. 2018 Sep 29;11:268. doi: 10.1186/s13068-018-1269-7 (PMC6162923; doi:10.1186/s13068-018-1269-7)
Supplement: Supplementary file 3 — Additional file 3: Table S3. Expression levels of known target genes dependent on Msn2/4p. [file 13068_2018_1269_MOESM3_ESM.docx]

**Table S3:** Expression levels of known target genes dependent on Msn2/4p.

| Gene name | Fold change in expression^a^ | Function | Description |
| --- | --- | --- | --- |
| *MSN2* | 0.36 | Stress-responsive transcriptional activator | Transcriptional activator |
| *MSN4* | 2.42 |  |  |
| Msn2/4p-dependent proteins^b^ | | | |
| *DDR48* | 4.99 | Heat shock, DNA damage or osmotic stress-responsive protein | - |
| *HSP26* | 3.15 | Heat shock protein | Protein folding chaperone |
| *HSP31* | 2.7 | Methylglyoxalase/heat shock protein | Protein folding chaperone |
| *GSY2* | 2.73 | Glycogen synthase | Carbohydrate metabolism |
| *CTT1* | 1.89 | Cytosolic catalase T | Oxidative stress defense |
| *ALD2* | 1.77 | Aldehydedehydrogenase | Carbohydrate metabolism |
| *ALD3* | 1.67 | Aldehydedehydrogenase | Carbohydrate metabolism |
| *MSC1* | 1.51 | Protein of unknown function | - |
| *HXK1* | 1.49 | Hexokinase isoenzyme 1 | Carbohydrate metabolism |
| *SSA3* | 0.40 | ATPase involved in protein folding and the response to stress | Protein folding chaperone |
| *TKL2* | 0.30 | Transketolase | Carbohydrate metabolism |

^a^Fold change is the ratio of the transcription level in evolved cells (XUSE) to that in control cells (XUS) (*p* < 0.05).

^b^[36, 61, 62]

**References**

61. Boy-Marcotte E, Perrot M, Bussereau F, Boucherie H, Jacquet M. Msn2p and Msn4p control a large number of genes induced at the diauxic transition which are repressed by cyclic AMP in *Saccharomyces cerevisiae.* J Bacteriol. 1998;180(5):1044- 52.

62. Gauci VJ, Beckhouse AG, Lyons V, Beh EJ, Rogers PJ, Dawes IW, Higgins VJ. Zinc starvation induces a stress response in *Saccharomyces cerevisiae* that is mediated by the Msn2p and Msn4p transcriptional activators*.* FEMS Yeast Res. 2009;9:1187- 95.
