# Supplementary figures and images for: Genomic and phenotypic characterization of a refactored xylose-utilizing Saccharomyces cerevisiae strain for lignocellulosic biofuel production
Source: Biotechnol Biofuels. 2018 Sep 29;11:268. doi: 10.1186/s13068-018-1269-7 (PMC6162923; doi:10.1186/s13068-018-1269-7)

### (A) XI-based pathway

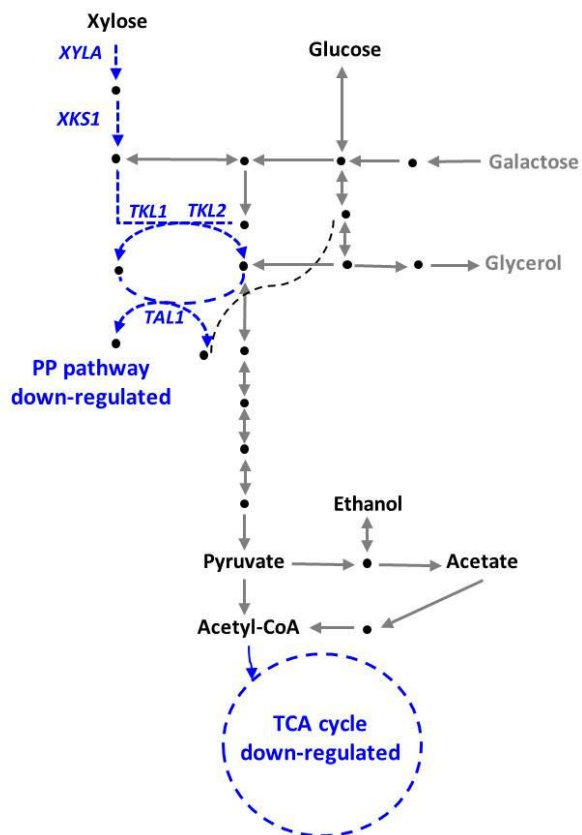

### (B) XR/XDH-based pathway

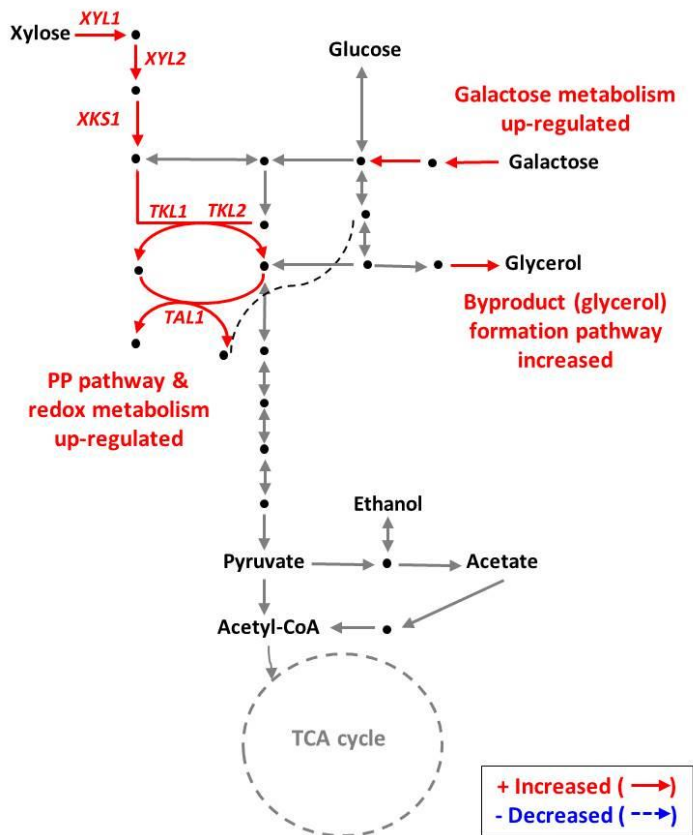

Supplement: Supplementary file 4 — Additional file 4: Figure S1. Comparison of the trends in the transcriptional changes in XI-based (a) and XR/XDH-based (b) strains during adaptive evolution on xylose. [file 13068_2018_1269_MOESM4_ESM.pdf]
